# Operations on the Superior Maxilla

**Published:** 1893-01

**Authors:** 


OPERATIONS ON THE SUPERIOR MAXILLA.—Pantaloni (*Arch. Prov. de Chir.*, 1892, No. 3) concludes that by the use of the position recommended by Rose in operations upon the superior maxilla, the use of the gag of O'Dwyer, the fixation of the tongue by forceps, and the hæmostatic use of tampons, perfect antiseptic conditions can be had without the annoyance of hemorrhage during the operation, while complete narcosis can be maintained throughout the operation by the interrupted method of chloroform anæsthesia. These methods, he believes, make unnecessary a previous tracheotomy and ligation of the external carotid, as also ligatures during or after the operation.

